# Dose-Dependent Neuroprotective Effect of Standardized Bee Venom Phospholipase A_2_ Against MPTP-Induced Parkinson’s Disease in Mice

**DOI:** 10.3389/fnagi.2019.00080

**Published:** 2019-04-05

**Authors:** Kyung Hwa Kim, Seung Young Lee, Jaekwon Shin, Jae-Taeg Hwang, Hat Nim Jeon, Hyunsu Bae

**Affiliations:** ^1^Department of Physiology, College of Korean Medicine, Kyung Hee University, Seoul, South Korea; ^2^Inist ST Co. Ltd., Seongnam-si, South Korea

**Keywords:** Parkinson’s disease, regulatory T cells, bee venom phospholipase A_2_, neuroinflammation, dose-dependent response

## Abstract

Parkinson’s disease (PD) is a chronic progressive neurodegenerative movement disorder characterized by the selective loss of dopaminergic neurons within the substantia nigra (SN). While the precise etiology of dopaminergic neuronal demise is elusive, multiple lines of evidence indicate that neuroinflammation is involved in the pathogenesis of PD. We have previously demonstrated that subcutaneous administration of bee venom (BV) phospholipase A_2_ (bvPLA_2_) suppresses dopaminergic neuronal cell death in a PD mouse model. In the present study, we established standardized methods for producing bvPLA_2_ agent isolated from crude BV at good manufacturing practice (GMP) facility. The therapeutic efficacy of purified bvPLA2 agent was examined in MPTP-induced PD mice. Importantly, administration of purified bvPLA_2_ in a dose-dependent manner reversed motor deficits in PD mice as well as inhibited loss of dopaminergic neurons within the SN of PD mice. The concentration-dependent action of standardized bvPLA_2_ appeared to be related to the induction of CD4^+^CD25^+^Foxp3^+^ regulatory T cells (Tregs), which, in part, inhibits T helper 1 (Th1) and Th17 polarization and suppresses microglial activation in PD mice. Taken together, these results suggest that standardized bvPLA_2_ purified from BV shows a neuroprotective effect against PD and thus has a potential target for treatment of PD.

## Introduction

Parkinson’s disease (PD) is known as the second most common neurodegenerative disease to affect about 3% of the population over the age of 65 (Lang and Lozano, 1998; de Lau et al., 2004). It is generally characterized by the selective loss of dopaminergic neurons as significant neuropathological hallmarks (Bertram and Tanzi, 2005). Until recently, several animal models have been developed to discover PD pathogenesis as well as search for neuroprotective therapeutic targets (Wang et al., 2015). Among all of these models, MPTP model has been widely used to initiate PD in animal models by penetrating blood-brain barrier (BBB) and destroying dopaminergic neurons in the substantia nigra (SN; Sakaguchi et al., 2008).

While the etiology of PD is unclear, a considerable body of research suggests that inflammatory responses play an important role in the development and progression of PD (Tufekci et al., 2012). Emerging evidence indicates the enhanced inflammatory responses, infiltration of T cells into the brain and glial cell activation are prominent features of PD (Wang et al., 2015). Recently, significant efforts recently have been focused on developing novel anti-inflammatory agents for PD treatment (Samii et al., 2009).

Bee venom (BV) consists of a complex mixture of peptides, enzymes, lipids and bioactive amines. Accumulating evidence suggests a wide range of pharmaceutical properties of BV. Accordingly, BV therapy has been developed to treat various diseases, including inflammatory diseases (Lee et al., 2014) and neurodegenerative diseases (Hossen et al., 2017).

One of the major components of BV is phospholipase A_2_ (PLA_2_), comprising approximately 10%–12% of the dry weight of the venom in the European honeybee, *Apis mellifera* (Habermann, 1972). The PLA2 derived from BV (bvPLA_2_) belongs to group III secretory PLA_2_ (sPLA_2_) that has been implicated in diverse cellular responses, such as signal transduction, host defense, blood coagulation, and pain relief (Hossen et al., 2016). Interestingly, several lines of evidence have indicated the therapeutic effect of bvPLA_2_ in neurodegenerative diseases, including prion disease (Jeong et al., 2011). Consistent with this finding, we previously reported the neuroprotective effect of bvPLA_2_ against neurodegenerative diseases including Alzheimer’s disease (Ye et al., 2016) and PD (Chung et al., 2015). The cellular action of bvPLA_2_ appeared to mainly suppress immune responses *via* stimulation of dendritic cells, ultimately enhancing the function of regulatory T cells which play an essential role in maintaining immune tolerance (Sakaguchi et al., 2008).

It appears that generating clinical-grade and sterile pharmaceutical products from BV is challenging, mostly related to identification, isolation, and purification of bioactive components from BV (Ameratunga et al., 1995; Lee et al., 2014). In the present study, we developed an effective strategy for therapeutic components based on bvPLA_2_ which were isolated and purified from BV at good manufacturing practice (GMP) facility. To evaluate the translational relevance, we tested the neuroprotective effects of the standardized bvPLA_2_ in MPTP-induced mouse model of PD. Additionally, dose-dependent effect of bvPLA_2_ isolated from BV on MPTP-induced PD mice was investigated to determine an optimal dose. Thus, the present study may shed new light on developing new therapeutic targets for PD to provide a basis for standardization and GMP of bvPLA_2_ drug.

## Materials and Methods

### Animals

All experiments were performed in accordance with the approved animal protocols and guidelines established by Kyung Hee University. Briefly, 7- to 8-week-old male C57BL/6J mice were purchased from The Jackson Laboratory (Bar Harbor, ME, USA). All mice were maintained under pathogen-free conditions on a 12-h light/dark cycle and temperature-controlled conditions, with food and water *ad libitum*.

### BvPLA_2_ Isolation, Preparation, Manufacturing, and Quality Management

A standardized BV PLA2 was prepared by Inist St Co. Limited (Eumseong-gun, South Korea). For isolation and purification, raw BV was purchased from Bee Venom Lab LLC (Tbilisi, GA, USA) and dissolved in high performance liquid chromatography (HPLC) grade water at a concentration of 1 mg/ml. Then the diluted samples were applied to PTFE membrane filter (pore size 0.45 μm; Sigma-Aldrich, St Louis, MO, USA). To reduce the volume for the subsequent steps, the filtered mixtures were further concentrated by a tangential flow filtration (TFF) system, fitted with Pellicon 3 devices with Ultracel-10kDa membrane (Merck Millipore, Billerica, MA, USA). For manufacturing, the purified bvPLA_2_ was dried with freeze-drying and was collected as a white powder. The bvPLA_2_ content was determined using HPLC system and then diluted to a concentration of 0.1 mg/ml. Undesired substances including allergen were removed by membrane filters (pore size 0.22 μm PVDF sterile membrane filter; Jet Bio-Filtration Co., Ltd, Guangzhou, China). The separation and detection were carried out on reversed-phase (RP)-HPLC system on a C18 column (pore size: 180 Å; Sigma-Aldrich, St Louis, MO, USA) using a Waters 2695 liquid chromatograph and a Waters 2489 UV-visible detector (Waters Corporation, Milford, MA, USA). The sample was chromatographed at 25°C at a flow rate of 2 ml/min. The elution was performed with a linear gradient of 0%–80% acetonitrile in 0.1% trifluoroacetic acid (TFA; Sigma-Aldrich, St Louis, MO, USA) and the elution profile was monitored at 220 nm. The area of the peak detected was used to measure the recovery of bvPLA_2_ and the separation profiles of purified bvPLA_2_ were compared with those of commercial standard bvPLA_2_ (Sigma-Aldrich, St Louis, MO, USA). The bioactivity of purified bvPLA_2_ was compared with inactive mutated recombinant bvPLA_2_-H34A (Lee et al., Manuscript submitted for publication). In order to measure bioactivity of purified bvPLA_2_, PLA_2_ activity was measured with EnzCheck PLA2 Assay Kit (Invitrogen, Carlsbad, CA, USA) according to the supplier’s instructions. All these procedures were carried out at an aseptic GMP facility. For quality management, the purity test was performed to ensure that there was no detectable heavy metals, insoluble particulate matter, endotoxins, or microbes. A quality control using endotoxin assays was performed by Charles River Laboratories Korea (Incheon, South Korea). Certificate of Analysis, which specifies the pyrogenicity of the endotoxin assessed by kinetic turbidimetric assay, was supplied. The commercial standard endotoxin was dissolved and diluted with Limulus amebocyte lysate (LAL) reagent water (LRW) and tris aminomethane buffer in order to determine the endotoxin level of purified standard bvPLA_2_. Changes in the bvPLA_2_ content were examined for 3 months in stability test. Hence, based on these observations, the purified bvPLA_2_ appeared to be appropriate as standardized bvPLA_2_.

### MPTP-Induced PD Mouse Model

1-Methyl-4-phenyl-1,2,3,6-tetrahydropyridine (MPTP; 20 mg/kg; Sigma-Aldrich, St Louis, MO, USA) was intraperitoneally (i.p.) administered to mice four times a day at 2 h intervals, inducing severe and persistent depletions of dopamine as previously described (Jackson-Lewis and Przedborski, 2007). During the experiment, mice were monitored for their physical condition and weight loss. The mortality rate of mice after MPTP injection was 0%–30% in each group and the animals surviving with <20% weight loss were included in the analysis.

### BvPLA_2_ Treatment

One day after the last MPTP injection, MPTP-injected mice were received with either bvPLA_2_ or phosphate buffered saline (PBS). For administration, bvPLA_2_ was dissolved in PBS and administered by once daily subcutaneous (s.c.) injections for six consecutive days in the concentration range of 0.01–0.5 mg/kg. Mice treated with commercial standard bvPLA_2_ (Sigma-Aldrich, St Louis, MO, USA) by s.c. injection at a dose of 0.5 mg/kg for 6 days was used to compare the effect of purified bvPLA_2_.

### Immunohistochemistry

Immunohistochemical analysis was carried out, as previously described with minor modifications (Chung et al., 2015). Briefly, the tissues were incubated with the relevant primary antibody [tyrosine hydroxylase (TH; Mukhopadhyay and Stahl, 1995), Iba1 (1:2,000, Wako Pure Chemic Industries, Osaka, Japan), and ED1 (1:500, Serotec, Oxford, UK)] at 4°C overnight. Then, the tissues were incubated with biotinylated goat anti-secondary antibody (1:200, Vector Laboratories, Burlingame, CA, USA) and horseradish peroxidase (HRP)-conjugated streptavidin-biotin complex (Vectastain Elite ABC kit; Vector Laboratories) and visualized with diaminobenzidine (DAB). The stained cells were analyzed under a bright field microscope (Nikon, Tokyo, Japan).

### Unbiased Stereological Estimation

Unbiased stereological estimation of the total number of TH-, ED1-, or H&E-positive cells was made using an optical fractionator as described previously, with minor modifications (West, 1993). In brief, the sections used for counting covered the entire SN from the rostral tip of the SN pars compacta (SNpc) to the caudal end of the SN pars reticulate (SNr). The counting was carried out using the Olympus CAST-Grid system (Olympus, Ballerup, Denmark). The counting frame was placed randomly on the first counting area and moved systematically over all counting areas until the entire delineated area was sampled. The total number of cells was calculated according to the optical fractionator equation (West et al., 1991).

### Flow Cytometry

Flow cytometric detection of regulatory T cells was performed using fluorescein isothiocyanate (FITC)-conjugated anti-mouse CD4 (clone GK1.5; eBioscience, San Diego, CA, USA), phycoerythrin (PE)-conjugated anti-mouse CD25 (clone PC61.5; eBioscience, San Diego, CA, USA), and Alexa Fluor 647 anti-mouse Foxp3 (clone MF23; BD Pharmingen™, San Jose, CA, USA). The single-cell splenocytes were washed with PBS and stained with FITC-conjugated anti-CD4 and PE-labeled anti-CD25 antibodies in staining buffer. The cells were subsequently fixed and stained with Alexa Fluor 647 anti-Foxp3 antibody overnight at 4°C in the dark. After washing, the cells were stored at 4°C in the dark for subsequent detection.

### Enzyme-Linked Immunosorbent Assay (ELISA)

To assess CD4^+^ T helper (Th) cell subsets from splenocytes, we measured cytokine production by enzyme-linked immunosorbent assay (ELISA). First, the splenocytes at a concentration of 1 × 10^7^ cells/ml were prepared from mice. Then, splenic CD4^+^ T cells were isolated by positive selection using anti-CD4 (L3T4) MicroBeads (Miltenyi Biotec, Bergisch Gladbach, Germany) according to the manufacturer’s instructions. The purified splenic CD4^+^ T cells were stimulated for 12 h with 50 ng/ml of phorbol myristate acetate (Sigma-Aldrich, St Louis, MO, USA) and 1,000 ng/ml of ionomycin (Sigma-Aldrich, St Louis, MO, USA). The supernatants were taken and the levels of IFN-γ, IL-4, and IL-17A were measured from these supernatants by ELISA kits (BD Biosciences, San Jose, CA, USA) according to the supplier’s instructions.

### Pole Test

The degree of bradykinesia of the mouse was measured by the pole test with a slight modification of a previous protocol (Ogawa et al., 1985). Briefly, a tube of ~50 cm in length and ~1 cm in diameter was wrapped in gauze and a wooden pole was attached to the top. The time at which the mice turned completely downward and the total time to climb down the pole were measured with a cut-off limit of 30 s. Each mouse was given five trials, and the average of the best three measurements was used as the result. Trials, where the mouse jumped or slid down the pole, were excluded.

### Statistical Analysis

Statistical analysis was performed using GraphPad Prism (v5.0; GraphPad) software. Each data was compared between conditions using unpaired *T*-test or one-way analysis of variance (ANOVA), or Kruskal-Wallis test followed by *post hoc* group comparisons. Data are expressed as the means ± standard error of the mean (SEM); *P* < 0.05 was considered significant.

## Results

### Standardization of the Manufacturing Process of Bee Venom PLA_2_

**Figure 1:**
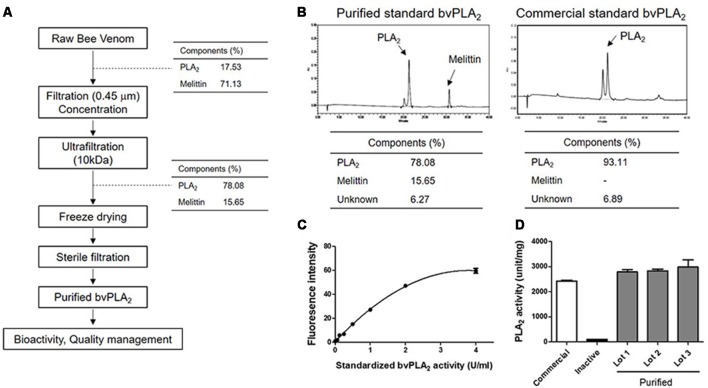
Developing methods for preparing bee venom phospholipase A_2_ (bvPLA_2_) standard reagent from BV. **(A)** Purification and manufacturing process of PLA_2_ from raw BV at a good manufacturing practice (GMP) facility. **(B)** Identification and determination of the purity of purified and commercial standard bvPLA_2_. **(C)** Standard curve of purified standard bvPLA_2_. **(D)** The activity of PLA_2_ from purified bvPLA_2_ isolated from BV (Lot 1–3), inactive recombinant mutant bvPLA_2_ (bvPLA_2_-H34A), and commercial bvPLA_2_. Combined results were plotted from at least two independent experiments. The data are expressed as the means ± standard error of the mean (SEM).

In the present study, we developed standardized methods for preparing bvPLA_2_ from active components of European honeybee, *Apis mellifera* (Figure 1A). To purify the crude BV, the extract was ultra-filtrated and subsequently concentrated to increase the yield of bvPLA_2_, as well as to remove undesirable products. Purified standard bvPLA_2_ were identified and separated using RP-HPLC system with a C18 column (Figure 1B). Commercial standard bvPLA_2_ was also used to identify the separation profiles of purified bvPLA_2_ and determine the content of purified of bvPLA_2_. Additionally, the bioactivity test was carried out to test specific PLA_2_ activity (Figures 1C,D). To ensure that purified bvPLA_2_ was safe, we performed the quality control tests, resulting in that it had an allowable endotoxin level approved by US Food and Drug Administration (FDA; Table 1). Therefore, based on these observations, we considered this purified bvPLA_2_ as properly manufactured and standardized agent.

**Table 1:** Endotoxin analysis of purified standard bee venom phospholipase A_2_ (bvPLA_2_).

Fold-dilution	Spike recovery (%)	CV (%)	Endotoxin value (EU/ml)
10	64	1.82	0.6915
50	98	0.83	0.3776
100	109	<0.01	0.5447
200	123	<0.01	0.6151

### BvPLA_2_ Improves Motor Activity in a Dose-Dependent Manner in MPTP-Induced PD Mice

**Figure 2:**
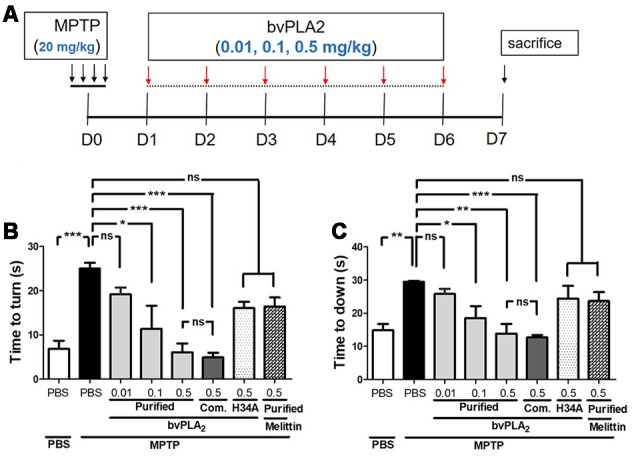
Dose-dependent neuroprotection of purified standard bvPLA_2_ on motor deficits in MPTP-injected mice. **(A)** Standard bvPLA_2_ purified from BV (0.01, 0.1, or 0.5 mg/kg), commercial standard bvPLA_2_ (Com. bvPLA_2_; 0.5 mg/kg), inactive recombinant mutant bvPLA_2_ (bvPLA_2_-H34A; 0.5 mg/kg), or standard melittin purified from BV (0.5 mg/kg) was administered to the mice for 6 days, beginning 1 day after MPTP injection. **(B,C)** The motor ability of mice was evaluated on a pole test on day 6 post-MPTP. Time to orient downward **(B)** and total time to descend **(C)** were measured. The data are expressed as the means ± SEM, *n* = 5–8 per group; *p < 0.05, **p < 0.01, ***p < 0.001, ns, not significant.

We first examined whether purified standard bvPLA_2_ protected behavioral deficits in PD mice. As shown in Figure 2A, purified bvPLA_2_ was given to MPTP-treated mice at a dose of 0.01, 0.1, or 0.5 mg/kg for six consecutive days, beginning 1 day after the last MPTP injection. On day 6 after MPTP treatment, MPTP-challenged mice took much longer than the control mice to turn downward (Figure 2B) and to descend the pole (Figure 2C), indicating basal ganglia-related movement disorders in MPTP-treated mice. Treatment of purified bvPLA_2_ significantly shortened the time to turn and to down in MPTP-treated mice. However, inactive mutant bvPLA_2_ (H34A) treatment induced no significant difference compared with MPTP-treated mice. No significant differences were detected between purified standard bvPLA_2_ group and commercial standard bvPLA_2_ group treated with the same dose (0.5 mg/kg). As observed, administration of purified standard bvPLA_2_ improved motor deficits induced by MPTP in a concentration-dependent manner.

### BvPLA_2_ Is a Regulator of Peripheral Regulatory T Cell Differentiation

Growing body of evidence supported the role of regulatory T cells in the disease progression of PD both in human PD patients (Chen et al., 2015) and animal models of PD (Reynolds et al., 2010). Here, we speculated that the action of purified standard bvPLA_2_ on improving sensorimotor function in PD mice may be related to the induction of Tregs in the periphery.

**Figure 3:**
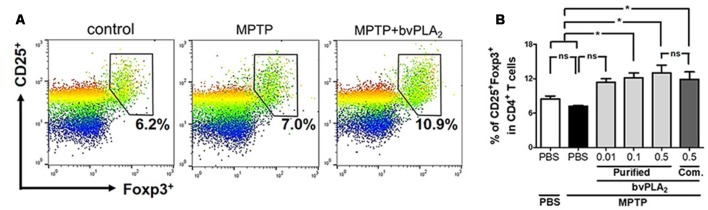
Dose-dependent effect of purified standard bvPLA_2_ on inducing differentiation of Treg cells in MPTP-treated mice. **(A,B)** On day 7 after MPTP treatment, CD4^+^CD25^+^Foxp3^+^ regulatory T cells in splenocytes were analyzed by flow cytometry. The percentage of CD25^+^Foxp3^+^ cells in CD4^+^ T cells were assessed in MPTP-treated mice with bvPLA_2_ treatment (purified bvPLA_2_: 0.01, 0.1, 0.5 mg/kg; commercial (Com.) bvPLA_2_: 0.5 mg/kg) for consecutive 6 days after 1 day of MPTP challenge. Combined results from two independent experiments with at least three mice per group were plotted. MPTP+bvPLA_2_ group: standard bvPLA_2_, purified from crude BV, was injected from Day 1 to Day 6 post-MPTP at a concentration of 0.5 mg/kg. The data are expressed as the means ± SEM; *p < 0.05, **p < 0.01, ***p < 0.001, ns, not significant.

To address our hypothesis, we first measured the cellular proportions of Tregs in MPTP mice after treatment with different doses of bvPLA_2_ (Figure 3). In fact, no significant difference in CD4^+^CD25^+^Foxp3^+^ Treg populations was detected between control and MPTP mice on 7 days after MPTP challenge (Figure 3B). Importantly, administration of bvPLA_2_ at a concentration of 0.1 and 0.5 mg/kg induced an increase in the number of splenic Tregs on MPTP-treated mice, when compared with mice treated with MPTP only. Whereas lower doses of bvPLA_2_ (0.01 mg/kg) induced no significant differences in the population of Treg cells. Similarly, commercial bvPLA_2_ (0.5 mg/kg) stimulated Treg induction in the periphery with no significant difference compared with purified standard bvPLA_2_ (0.5 mg/kg). All these findings support the notion that bvPLA_2_ induces the expansion of CD4^+^CD25^+^Foxp3^+^ Treg in the periphery in PD, which can suppress the inflammatory response.

### BvPLA_2_ Inhibits the Expansion of Th1 and Th17 Effector Cells That Are Associated With PD

**Figure 4:**
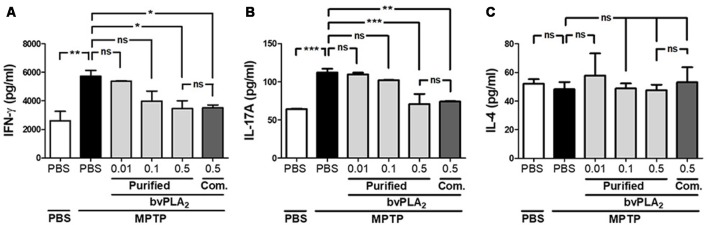
Dose-dependent inhibitory effect of purified standard bvPLA_2_ on Parkinson’s disease (PD)-related Th1 and Th17 polarization. Th1, Th2, and Th17-polarizing conditions were analyzed by enzyme-linked immunosorbent assay (ELISA) of Th1 cytokine (IFN-γ; **A**), Th2 cytokine (IL-4; **C**), and Th17 cytokine (IL-17A; **B**) from the supernatants of splenic CD4^+^ T cells in MPTP-injected mice treated with purified standard bvPLA_2_ (0.01, 0.1, or 0.5 mg/kg) or commercial standard bvPLA_2_ (Com. bvPLA_2_; 0.5 mg/kg). The data are expressed as the means ± SEM, *n* = 3–5 per group; *p < 0.05, **p < 0.01, ***p < 0.001, ns, not significant.

Since the balance of CD4^+^ T cell subsets is highly correlated with disease activity in PD (Chen et al., 2015), we further explored whether T helper subset balance was altered in PD. As shown in Figure 4, we assessed the differentiation of T helper (Th) cells based on their cytokine signature: IFN-γ-secreting Th1, IL-4-secreting Th2 and IL-17A-producing Th17 cells (Zhu et al., 2010). Obviously, MPTP treatment increased IFN-γ-secreting Th1 cells, but not IL-4-secreting Th2, indicating a shifted Th1/Th2 balance towards Th1 in PD mice (Figure 4). Additionally, we asked whether purified standard bvPLA_2_ could reverse the altered balance of Th subsets in PD mice. Impressively, bvPLA_2_ treatment significantly reduced the secretion of the two pro-inflammatory cytokines IFN-γ and IL-17A in a dose-dependent fashion (Figure 4). Commercial bvPLA_2_ injection (0.5 mg/kg) also exhibited a similar suppressive effect on Th1- and Th17-polarizing cytokines associated with PD. These results showed that specific CD4^+^ T subsets, including Th1 and Th17 cells, were markedly differentiated in PD induced by MPTP. Impressively, purified standard bvPLA_2_ in a dose-dependent manner suppressed MPTP-mediated imbalance of CD4^+^ T cell subsets.

### BvPLA_2_ Protects Dopaminergic Neurons Against MPTP Neurotoxicity in a Concentration-Dependent Manner

**Figure 5:**
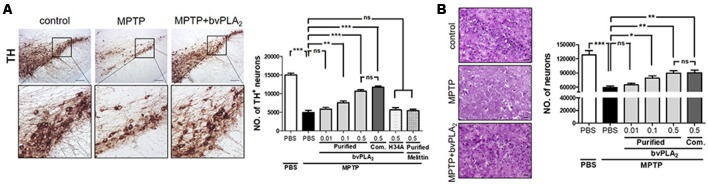
Dose-dependent suppressive effect of purified standard bvPLA_2_ on neuronal death of MPTP-challenged mice. Immunohistochemical staining for tyrosine hydroxylase (TH; **A**) or H&E **(B)** was carried out on MPTP mice with the treatment of standard bvPLA_2_ purified from crude BV (0.01, 0.1, or 0.5 mg/kg) or commercial standard bvPLA_2_ (Com. bvPLA_2_; 0.5 mg/kg) on day 7 post-MPTP. Representative images of immunohistochemical staining for TH-positive or H&E stained cells in substantia nigra (SN) are shown. The number of stained cells was quantified by using an unbiased stereology. High-magnification images of boxed regions on TH-stained sections are also shown. MPTP+bvPLA_2_ group: standard bvPLA_2_, purified from crude BV, was injected from Day 1 to Day 6 post-MPTP at a concentration of 0.5 mg/kg. The data are expressed as the means ± SEM. *n* = 4–8 per group; *p < 0.05, **p < 0.01, ***p < 0.001, ns, not significant. Scale bar: 100 μm **(A)**; 50 μm **(B)**.

We further evaluated whether purified standard bvPLA_2_ exhibited a dose-dependent neuroprotection against neuronal degeneration of PD. Expectedly, the immunohistochemistry data revealed apoptotic cell death in H&E stained and loss of dopaminergic neurons in TH stained sections of MPTP-challenged mice (Figure 5). In contrast, there was a dramatic neuronal protection in SN of MPTP mice following bvPLA_2_ treatment in a dose-dependent manner. When administered at a comparative lower dose (0.01 mg/kg), bvPLA_2_ administration did not significantly rescue MPTP-induced neuronal loss. Similar results were observed in MPTP mice given with commercial bvPLA_2_ at a dose of 0.5 mg/kg. These data indicate that purified bvPLA_2_ effectively attenuated the loss of dopaminergic neurons associated with PD in a concentration-dependent way.

### BvPLA_2_ Suppresses Microglial Activation Caused by MPTP in Mice in a Dose-Dependent Manner

**Figure 6:**
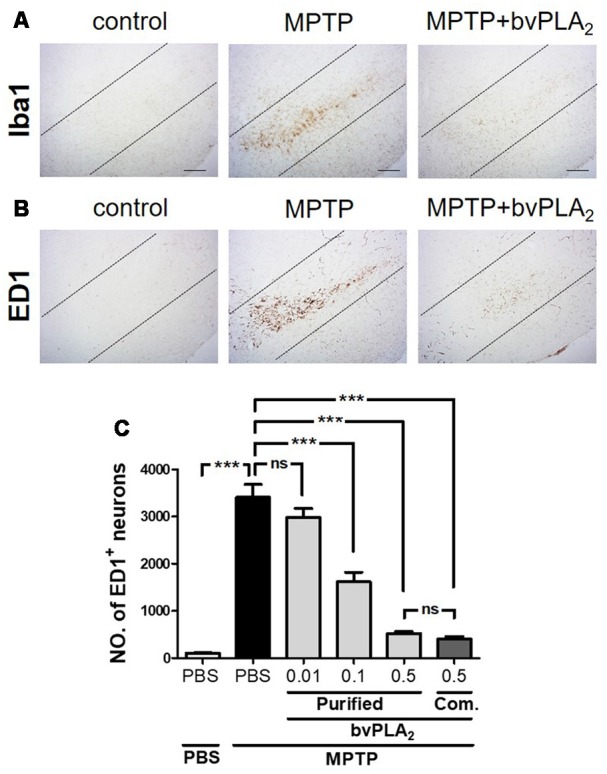
Dose-dependent inhibitory effect of purified standard bvPLA_2_ on microglial activation in the MPTP mouse model of PD. Immunohistochemical analysis was performed on MPTP-challenged mice treated with standard bvPLA_2_ purified from crude BV (0.01, 0.1, or 0.5 mg/kg), or commercial standard bvPLA_2_ (Com. bvPLA_2_; 0.5 mg/kg) for consecutive 6 days. Representative images of sections containing Iba1- **(A)** or ED1-positive **(B)** neurons are displayed and dotted lines indicate SN pars compacta (SNpc). MPTP+bvPLA_2_ group: standard bvPLA_2_, purified from crude BV, was injected from Day 1 to Day 6 post-MPTP at a concentration of 0.5 mg/kg. **(C)** Unbiased stereological estimation for ED1-postive neurons was performed in SN region. The data are expressed as the means ± SEM. *n* = 3–5 per group; ***p < 0.001, ns, not significant. Scale bar: 100 μm.

Mounting evidence has demonstrated that microglial activation may play a key role in amplifying the neuroinflammatory response, which can exacerbate dopaminergic neuronal death (Kim and Joh, 2006). Hence, we examined the expression levels of microglia markers, such as Iba1 and ED1, to explore whether purified standard bvPLA_2_ could directly suppress the activation of microglia. As shown in Figure 6A, the level of Iba1-positive microglia was markedly increased in the SN of MPTP mice at 7 days after MPTP post-injection, whereas bvPLA_2_ administration clearly reduced the expression of Iba1 in PD mice a concentration-dependent manner. Similarly, MPTP caused a significant increase in ED1-positive neuronal cells compared with the control group, while administration of purified bvPLA_2_ markedly reduced the number of ED1^+^ microglia in the brains of MPTP-challenged mice (Figures 6B,C). The effect of commercial standard bvPLA_2_ (0.5 mg/kg) appeared to be similar to that of 0.5 mg/kg of purified bvPLA_2_, showing no difference between the two groups. All these findings support the notion that purified bvPLA_2_ inhibits microglial activation in MPTP-treated mice in a dose-dependent manner.

## Discussion

In the present study, we developed an effective method of bvPLA_2_ purification to create a valid and safe standardized medicine. Using MPTP animal models, we further demonstrated the therapeutic potential of purified standard bvPLA_2_ against PD. Additionally, we identified the dose-dependent neuroprotective effect of purified standard bvPLA_2_ on PD mice.

Growing evidence suggests that neuroinflammation is involved in the development and progression of PD (Wang et al., 2015). It is generally suggested that nerve damage in the pathogenesis of PD can be aggravated by the activated microglia producing an excess of pro-inflammatory cytokines. In contrast, some recent studies have proposed that activated microglia, especially M2 microglia, can be beneficial, at least in the early phase of the neurodegeneration process (Hu et al., 2012). Additionally, a growing body of evidence suggests that neuroinflammatory processes can act as a double-edged sword in diverse pathologic conditions linked to neurodegenerative diseases (Wang et al., 2015). Hence, when identifying a novel anti-inflammatory drug for treating neurodegenerative disease, though evaluation to designate a therapeutic window at a minimum effective dose is necessary.

To our knowledge, the present study was the first to present manufacturing process of bvPLA_2_ from crude BV, as well as to assess its therapeutic efficacy for treatment of PD. We developed effective strategies to isolate and purified bvPLA_2_ from active components of crude BV at GMP facilities. The separated bvPLA_2_ displayed bioactivity with showing a specific activity of about 2,000 unit/mg. In order to evaluate translational relevance and therapeutic potential of purified bvPLA_2_, we examined the therapeutic effect of purified bvPLA_2_ by injecting three different doses (0.01, 0.1, 0.5 mg/kg) on MPTP-challenged PD mice. Indeed, it is known that high doses of bvPLA_2_ (2.5 mg/kg) can cause a variety of allergic reactions, while relatively low doses of bvPLA_2_ (0.25 mg/kg) induce protective effects against inflammatory conditions (Palm et al., 2013). Previously, we also observed that relatively low doses of bvPLA_2_ (0.2–0.5 mg/kg) could induce anti-inflammatory effects in murine models of atopic dermatitis (Jung et al., 2017) and asthma (Park et al., 2015). Hence, we assumed the biological function of bvPLA_2_ may differ in a dose-dependent manner, reflecting that at low doses bvPLA_2_ act more generally to reduce neuroinflammation. Further studies are required to clarify the paradoxical effect and dose-dependent activity of bvPLA_2_.

In this study, we found a dose-dependent neuroprotective effect of purified standard bvPLA_2_ containing ~78% of PLA_2_ and ~15% of melittin. In line with the experiments with purified bvPLA2, we also examined whether melittin caused a neuroprotective effect against PD by isolated from crude BV with high purity (98.5% of melittin). Administration of purified melittin did not induce any neuroprotective effect on PD mice. Additionally, the inactive mutant of bvPLA_2_ (bvPLA_2_-H34A) caused an inhibitory effect on the neuronal death and motor deficits in MPTP-challenged mice. Melittin is a major component of BV, comprising around 50% of dry venom. Recently, many studies have reported the therapeutic effects of melittin against cancers, neurodegenerative diseases and chronic inflammatory diseases including rheumatism (Park et al., 2004; Yang et al., 2011; Rady et al., 2017). It has been suggested that the biological activity of bvPLA_2_ can be enhanced by melittin (Mingarro et al., 1995; Shen et al., 2010). Certainly, more detailed sets of experimental investigations are needed to verify its potential usefulness in the future applications.

The initial study regarding the involvement of inflammation in PD progression over 30 years ago clearly reported activated microglia in the SN of a PD patient (McGeer et al., 1988). Since then accumulated studies support the role of activated microglia and increased inflammatory mediators in the pathology of PD (Collins et al., 2012). In particular, accumulating evidence suggests the important role of adaptive immune system in the development and progression of PD (Ferrari and Tarelli, 2011). Importantly, the cytotoxic CD4^+^ T cells have been discovered in the SN of PD patients and animal PD models (Brochard et al., 2009; Stone et al., 2009). Indeed, the adaptive immune system would modulate neuroinflammatory response *via* regulation of microglia activation (Tansey and Goldberg, 2010). In this study, we observed the inhibitory effect of bvPLA_2_ isolated and purified from BV on dopaminergic cell loss of MPTP mice. Impressively, induction of immunosuppressive regulatory T cells was observed in MPTP mice treated with purified standard bvPLA_2_. These results suggest that bvPLA_2_ may exert anti-inflammatory action *via* activation of Treg cells, which can suppress the inflammatory process by targeting cytotoxic CD4^+^ T cells.

In summary, we standardized the purification process to provide standard bvPLA_2_ from active component of crude BV and observed dose-dependent therapeutic effect of standard bvPLA_2_ on MPTP-induced PD mice. Based on our results, it appears that bvPLA_2_ is a potent drug that can promote the survival of dopaminergic neurons, suggesting a novel therapeutic candidate as an add-on to conventional dopaminergic substitution treatments.

## Ethics Statement

All experiments were performed in accordance with the approved animal protocols and guidelines established by Kyung Hee University.

## Author Contributions

KK and HB conceived and designed the project. KK, SL, JS, and HJ performed the experiments and analyzed the data. J-TH was in charge of standardization of the manufacturing process of bvPLA_2_ from crude BV. The manuscript was written by KK and HB.

## Conflict of Interest Statement

J-TH was employed by company Inist ST Co. Ltd.

The remaining authors declare that the research was conducted in the absence of any commercial or financial relationships that could be construed as a potential conflict of interest.

## References

[B1] Ameratunga R. V. Hawkins R. Prestidge R. Marbrook J. (1995). A high efficiency method for purification and assay of bee venom phospholipase A2. Pathology 27, 157–160. 10.1080/00313029500169782 7567144

[B2] Bertram L. Tanzi R. E. (2005). The genetic epidemiology of neurodegenerative disease. J. Clin. Invest. 115, 1449–1457. 10.1172/jci24761 15931380 PMC1137006

[B3] Brochard V. Combadiere B. Prigent A. Laouar Y. Perrin A. Beray-Berthat V.. (2009). Infiltration of CD4^+^ lymphocytes into the brain contributes to neurodegeneration in a mouse model of Parkinson disease. J. Clin. Invest. 119, 182–192. 10.1172/JCI36470 19104149 PMC2613467

[B4] Chen Y. Qi B. Xu W. Ma B. Li L. Chen Q.. (2015). Clinical correlation of peripheral CD4+cell subsets, their imbalance and Parkinson’s disease. Mol. Med. Rep. 12, 6105–6111. 10.3892/mmr.2015.4136 26239429

[B5] Chung E. S. Lee G. Lee C. Ye M. Chung H. S. Kim H.. (2015). Bee venom phospholipase A2, a novel Foxp3+ regulatory T cell inducer, protects dopaminergic neurons by modulating neuroinflammatory responses in a mouse model of Parkinson’s disease. J. Immunol. 195, 4853–4860. 10.4049/jimmunol.1500386 26453752

[B6] Collins L. M. Toulouse A. Connor T. J. Nolan Y. M. (2012). Contributions of central and systemic inflammation to the pathophysiology of Parkinson’s disease. Neuropharmacology 62, 2154–2168. 10.1016/j.neuropharm.2012.01.028 22361232

[B7] de Lau L. M. Giesbergen P. C. de Rijk M. C. Hofman A. Koudstaal P. J. Breteler M. M. (2004). Incidence of parkinsonism and Parkinson disease in a general population: the Rotterdam study. Neurology 63, 1240–1244. 10.1212/01.wnl.0000140706.52798.be 15477545

[B8] Ferrari C. C. Tarelli R. (2011). Parkinson’s disease and systemic inflammation. Parkinsons. Dis. 2011:436813. 10.4061/2011/436813 21403862 PMC3049348

[B9] Habermann E. (1972). Bee and wasp venoms. Science 177, 314–322. 10.1126/science.177.4046.314 4113805

[B10] Hossen M. S. Ali M. Y. Jahurul M. H. A. Abdel-Daim M. M. Gan S. H. Khalil M. I. (2017). Beneficial roles of honey polyphenols against some human degenerative diseases: a review. Pharmacol. Rep. 69, 1194–1205. 10.1016/j.pharep.2017.07.002 29128800

[B11] Hossen M. S. Shapla U. M. Gan S. H. Khalil M. I. (2016). Impact of bee venom enzymes on diseases and immune responses. Molecules 22:E25. 10.3390/molecules22010025 28035985 PMC6155781

[B12] Hu X. Li P. Guo Y. Wang H. Leak R. K. Chen S.. (2012). Microglia/macrophage polarization dynamics reveal novel mechanism of injury expansion after focal cerebral ischemia. Stroke 43, 3063–3070. 10.1161/strokeaha.112.659656 22933588

[B13] Jackson-Lewis V. Przedborski S. (2007). Protocol for the MPTP mouse model of Parkinson’s disease. Nat. Protoc. 2, 141–151. 10.1038/nprot.2006.342 17401348

[B14] Jeong J. K. Moon M. H. Bae B. C. Lee Y. J. Seol J. W. Park S. Y. (2011). Bee venom phospholipase A2 prevents prion peptide induced-cell death in neuronal cells. Int. J. Mol. Med. 28, 867–873. 10.3892/ijmm.2011.730 21701769

[B15] Jung K. H. Baek H. Kang M. Kim N. Lee S. Y. Bae H. (2017). Bee venom phospholipase A2 ameliorates house dust mite extract induced atopic dermatitis like skin lesions in mice. Toxins 9:E68. 10.3390/toxins9020068 28218721 PMC5331447

[B16] Kim Y. S. Joh T. H. (2006). Microglia, major player in the brain inflammation: their roles in the pathogenesis of Parkinson’s disease. Exp. Mol. Med. 38, 333–347. 10.1038/emm.2006.40 16953112

[B17] Lang A. E. Lozano A. M. (1998). Parkinson’s disease. First of two parts. N. Engl. J. Med. 339, 1044–1053. 10.1056/NEJM199810083391506 9761807

[B19] Lee J. A. Son M. J. Choi J. Jun J. H. Kim J. I. Lee M. S. (2014). Bee venom acupuncture for rheumatoid arthritis: a systematic review of randomised clinical trials. BMJ Open 4:e006140 10.1136/bmjopen-2014-006140 PMC4225238 25380812

[B21] McGeer P. L. Itagaki S. Boyes B. E. McGeer E. G. (1988). Reactive microglia are positive for HLA-DR in the substantia nigra of Parkinson’s and Alzheimer’s disease brains. Neurology 38, 1285–1291. 10.1212/wnl.38.8.1285 3399080

[B22] Mingarro I. Pérez-Payá E. Pinilla C. Appel J. R. Houghten R. A. Blondelle S. E. (1995). Activation of bee venom phospholipase A2 through a peptide-enzyme complex. FEBS Lett. 372, 131–134. 10.1016/0014-5793(95)00964-b 7556634

[B23] Mukhopadhyay A. Stahl P. (1995). Bee venom phospholipase A2 is recognized by the macrophage mannose receptor. Arch. Biochem. Biophys. 324, 78–84. 10.1006/abbi.1995.9926 7503563

[B24] Ogawa N. Hirose Y. Ohara S. Ono T. Watanabe Y. (1985). A simple quantitative bradykinesia test in MPTP-treated mice. Res. Commun. Chem. Pathol. Pharmacol. 50, 435–441. 3878557

[B25] Palm N. W. Rosenstein R. K. Yu S. Schenten D. D. Florsheim E. Medzhitov R. (2013). Bee venom phospholipase A2 induces a primary type 2 response that is dependent on the receptor ST2 and confers protective immunity. Immunity 39, 976–985. 10.1016/j.immuni.2013.10.006 24210353 PMC3852615

[B27] Park S. Baek H. Jung K. H. Lee G. Lee H. Kang G. H.. (2015). Bee venom phospholipase A2 suppresses allergic airway inflammation in an ovalbumin-induced asthma model through the induction of regulatory T cells. Immun. Inflamm. Dis. 3, 386–397. 10.1002/iid3.76 26734460 PMC4693726

[B26] Park H. J. Lee S. H. Son D. J. Oh K. W. Kim K. H. Song H. S.. (2004). Antiarthritic effect of bee venom: inhibition of inflammation mediator generation by suppression of NF-kappaB through interaction with the p50 subunit. Arthritis Rheum. 50, 3504–3515. 10.1002/art.20626 15529353

[B28] Rady I. Siddiqui I. A. Rady M. Mukhtar H. (2017). Melittin, a major peptide component of bee venom and its conjugates in cancer therapy. Cancer Lett. 402, 16–31. 10.1016/j.canlet.2017.05.010 28536009 PMC5682937

[B29] Reynolds A. D. Stone D. K. Hutter J. A. Benner E. J. Mosley R. L. Gendelman H. E. (2010). Regulatory T cells attenuate Th17 cell-mediated nigrostriatal dopaminergic neurodegeneration in a model of Parkinson’s disease. J. Immunol. 184, 2261–2271. 10.4049/jimmunol.0901852 20118279 PMC2824790

[B30] Sakaguchi S. Yamaguchi T. Nomura T. Ono M. (2008). Regulatory T cells and immune tolerance. Cell 133, 775–787. 10.1016/j.cell.2008.05.009 18510923

[B31] Samii A. Etminan M. Wiens M. O. Jafari S. (2009). NSAID use and the risk of Parkinson’s disease: systematic review and meta-analysis of observational studies. Drugs Aging 26, 769–779. 10.2165/11316780-000000000-00000 19728750

[B32] Shen L. R. Ding M. H. Zhang L. W. Zhang W. G. Liu L. Li D. (2010). Expression of a bee venom phospholipase A2 from Apis cerana cerana in the baculovirus-insect cell. J. Zhejiang Univ. Sci. B 11, 342–349. 10.1631/jzus.b0900254 20443212 PMC2865836

[B33] Stone D. K. Reynolds A. D. Mosley R. L. Gendelman H. E. (2009). Innate and adaptive immunity for the pathobiology of Parkinson’s disease. Antioxid. Redox Signal. 11, 2151–2166. 10.1089/ARS.2009.2460 19243239 PMC2788126

[B34] Tansey M. G. Goldberg M. S. (2010). Neuroinflammation in Parkinson’s disease: its role in neuronal death and implications for therapeutic intervention. Neurobiol. Dis. 37, 510–518. 10.1016/j.nbd.2009.11.004 19913097 PMC2823829

[B35] Tufekci K. U. Meuwissen R. Genc S. Genc K. (2012). Inflammation in Parkinson’s disease. Adv. Protein Chem. Struct. Biol. 88, 69–132. 10.1016/B978-0-12-398314-5.00004-0 22814707

[B36] Wang Q. Liu Y. Zhou J. (2015). Neuroinflammation in Parkinson’s disease and its potential as therapeutic target. Transl. Neurodegener. 4:19. 10.1186/s40035-015-0042-0 26464797 PMC4603346

[B37] West M. J. (1993). New stereological methods for counting neurons. Neurobiol. Aging 14, 275–285. 10.1016/0197-4580(93)90112-o 8367009

[B38] West M. J. Slomianka L. Gundersen H. J. (1991). Unbiased stereological estimation of the total number of neurons in thesubdivisions of the rat hippocampus using the optical fractionator. Anat. Rec. 231, 482–497. 10.1002/ar.1092310411 1793176

[B39] Yang E. J. Kim S. H. Yang S. C. Lee S. M. Choi S. M. (2011). Melittin restores proteasome function in an animal model of ALS. J. Neuroinflammation 8:69. 10.1186/1742-2094-8-69 21682930 PMC3142224

[B40] Ye M. Chung H. S. Lee C. Yoon M. S. Yu A. R. Kim J. S.. (2016). Neuroprotective effects of bee venom phospholipase A2 in the 3xTg AD mouse model of Alzheimer’s disease. J. Neuroinflammation 13:10. 10.1186/s12974-016-0476-z 26772975 PMC4715334

[B41] Zhu J. Yamane H. Paul W. E. (2010). Differentiation of effector CD4 T cell populations *. Annu. Rev. Immunol. 28, 445–489. 10.1146/annurev-immunol-030409-101212 20192806 PMC3502616

